# Contrasting Partners’ Traits of Generalized and Specialized Species in Flower-Visitation Networks

**DOI:** 10.1371/journal.pone.0150824

**Published:** 2016-03-03

**Authors:** Rocío Castro-Urgal, Anna Traveset

**Affiliations:** Institut Mediterrani d’Estudis Avançats (CSIC-UIB), Esporles, Mallorca, Balearic Islands, Spain; University of Northampton, UNITED KINGDOM

## Abstract

Much ecological research has focused on trying to understand why species are generalized or specialized in their interactions and how networks develop in a certain environment. It is now well known that traits such as phenology and abundance of a species are important determinants of its generalization level (i.e., number of different interactions or links to other species). Less information is available, however, on whether generalized and specialized species differ in particular traits of their interacting partners. Such partners might differ, for instance, in abundance and/or in the diversity of functional groups they belong to. Moreover, species might exhibit shifts through time (e.g., flowering season) in their partners’ traits, though we know close to nothing on whether these changes do indeed occur. Assessing how such network links in both types of species are established is important for a better understanding of how different types of disturbance can affect community dynamics. Using data from four quantitative flower-visitation networks and independent measures of flower availability obtained when recording interactions, we test for such differences between species which have been previously categorized according to two specialization indexes: (1) number of partners (links), also named linkage level; this is a qualitative index and (2) complementary specialization *d’*, named here selectiveness level; this is a quantitative index. We found that: (1) species with low linkage levels mainly interact with common species in the community whereas generalized species interact with a greater heterogeneity of partner’s abundances and functional richness, (2) both selective and opportunistic species (with high and low *d’*, respectively) interact with a similarly high functional richness (number of functional groups or families) of partners, and (3) generalized species are the only ones showing shifts along the season in their partners’ traits, driven by changes in community species composition. The risk of extinction in front of a disturbance is generally expected to be highest for specialized species (with few partners) and selective species (which visit non-abundant or scarce partners). However, our findings show that by linking to abundant and/or to functionally diverse partners, respectively, these species may be maintained in the community and be less vulnerable to disturbances.

## Introduction

Specialization in plant-pollinator interactions has been the focus of much research interest ever since Darwin [1]. Despite this, a non-ambiguous definition of specialization in pollination is still non-existent [2–4]. What seems clear is that specialization and generalization are context-dependent concepts: species may have different levels of specialization in varying locations, being generalized species in a poor pollinator environment but specialized in a rich pollinator environment. Moreover, they are the extremes of a gradient continuum [5]. The quantification of ecological specialization is also highly dependent on the data used, the organism studied, and the ecological mechanism of interest (e.g. behavior specialization vs. specialization for habitat) [6].

The study of plant-pollinator interactions has moved, in the last decades, from focusing on species pairs to the entire community, especially due to the development of complex network analysis techniques used in a multitude of research disciplines [7–9]. This new approach has promoted a better understanding of complex interactions between mutualistic partners and has allowed gathering evidence that moderate generalization is more the rule rather than the exception [5]. The first studies on pollination (and other mutualistic) interactions implementing this network approach were based on presence/absence interactions between species, giving each interaction the same weight [10–13]. In such studies, specialization is defined qualitatively as the species’ total number of interactions (i.e. species linkage level, *L*), so species linking to a high number of partners are considered generalized (e.g. one pollinator visiting a wide array of plant species) whereas species with low number of partners are specialized (e.g. one pollinator species visiting only one plant species). However, a plant pollinated by ten species of moths, for example, could be considered less generalized and therefore more vulnerable to disturbance than another plant pollinated by five species belonging to different pollinator functional groups. It was soon widely recognized by ecologists that this qualitative measure of generalization was limited as it fails to describe the strong heterogeneity in the frequency and availability of interaction partners and it is deeply dependent on network size.

The subsequent use of quantitative data to describe interaction strength between partners led to the emergence of a new concept of specialization [14], the species-level complementary specialization index (*d’*), based on Shannon diversity. This index takes into account not only the number of partners but also their availability in the community. Thus, it can be considered as an index of selectiveness (term used hereafter). A pollinator that visits a plant species proportionally to its availability in the community is considered opportunistic whereas one that visits rare plants disproportionately more than common ones is considered as selective. Likewise, an opportunistic plant is visited by pollinators proportionally to their availability whereas a selective plant is visited disproportionately more by rare than by common pollinators.

Several studies have explored the traits that contribute to the generalization level in flower-visitation networks. Traits such as phenology and abundance [15–21], flower color [22] or flower and insect morphology [23] have been shown to influence the number of different interactions a species can have. However, much less is known on whether the partners’ traits differ between generalized and specialized species and between selective and opportunistic species. The traits or mechanisms regulating interactions between species can actually be considered the “microstructure” of a network [24, 25]. The traits of species to which other species link to, for instance their abundance or their functional richness (here defined as the number of functional groups for flower visitors or the number of families for plants) are probably important determining such microstructure and their degree of vulnerability to disturbances.

**Fig 1:**
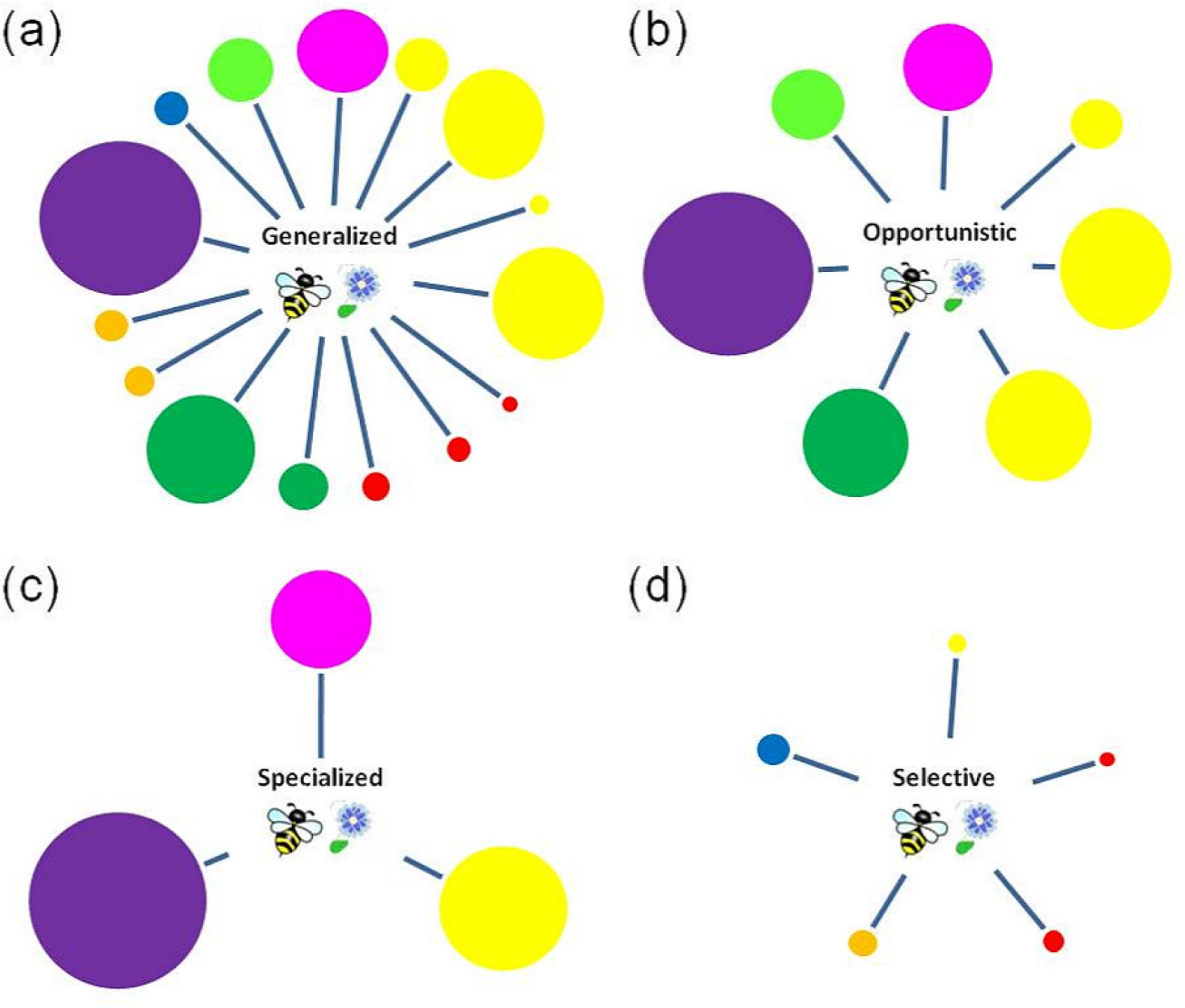
Representation of our hypotheses of species partners’ traits depending on the degree of specificity. (a) generalized, (b) opportunistic, (c) specialized and (d) selective species. Each circle represents a partner (plant or flower-visitor), whereas its size represents the abundance in the community. The variety of colors represents the functional richness of species with which it interact (families in the case of plants and functional groups in the case of flower-visitors).

In the present study, we are interested in investigating whether the partners’ traits of generalized and opportunistic species differ from those of specialized and selective species. Specifically, we aim at testing the following hypotheses: (1) to avoid risk of extinction, specialized species interact mainly with the most abundant species in the community. By contrast, (2) generalized species interact with species showing a greater heterogeneity of abundances (lower evenness of partners’ abundances). Moreover, (3) specialized and selective species should interact with a rich assemblage of partners because, in case of partners’ decline (e.g. an insect group or an entire plant family), they can interact with partners from other functional groups. (4) Generalized and opportunistic species show a high heterogeneity of partners’ functional richness which makes them more resistant against the extinction of a particular functional group. The four hypotheses are depicted in Fig 1.

The first hypothesis is related to network properties describing the overall patterns of interactions, such as nestedness and interaction asymmetry [13, 26, 27].

The temporal dynamics of species interactions needs also to be considered to better understand the microstructure of plant–pollinator communities. Partners’ traits may change along time (e.g. season) as interactions between plants and their flower visitors vary within and across seasons reflecting changes in community composition [17, 28–31]. Therefore, we might further expect specialized and selective species to be more constant in their partners’ traits through time than generalized and opportunistic species, which might be more variable following changes in species composition and abundances.

In order to test these hypotheses, we used data from four different communities for which we built quantitative flower-visitation networks, gathering independent measures of flower availability.

## Materials and Methods

### Ethics Statement

Servei de Proteccio´ d’Espècies, Espais de Natura Balear (Conselleria d’Agricultura, Medi Ambient i Territori) and Cabildo of Lanzarote provided permission to work at the study sites.

### Study sites and sampling procedure

Flower visitation networks were constructed from observations carried out in four coastal communities, two on Mallorca, Balearic Islands (Son Bosc and Cala Mesquida, SB and CM hereafter) and two on Lanzarote, Canary Islands (Caletón Blanco and Las Conchas, CB and LC hereafter) (see further description of the communities in [32]). The study was conducted in April-July 2010 on Mallorca and in January-April 2011 on Lanzarote, which covered the entire flowering spring season for both islands. Sampling started early in the year on Lanzarote because of the tight association between rainfall and plant flowering on this dry island.

Flower density was estimated every two weeks at each community by counting all open flowers of each plant observed along each of the ten (50 x 2 m^2^) permanent belt transects established in SB and in each of the 30 (0.5 x 0.5 m^2^) random plots located in the other three communities. Random plots instead of transects were used in these sites to capture the high heterogeneity of each area. Flower density was calculated for each species as the number of open flowers divided by the total area surveyed. For species with tightly clustered inflorescences (e.g. the capitula of Asteraceae), each inflorescence was scored as an individual flower. Flower-visitor abundance was estimated as the total number of individuals observed visiting flowers. Although the ideal would be to have also independent measures of flower-visitor abundance, this is usually not possible owing to the difficulties of tracking different types of insects.

On each census day at each community, all plants in bloom were observed the same amount of time to prevent the bias of finding more interactions in more abundant plant species. Censuses were performed once or twice a week at each site. We made randomized focal censuses, i.e. observing individuals of each flowering plant species at a time, between 10:00 and 17:00 h on sunny and low-wind days. Interactions were recorded from a distance of approximately 1 m from the focal plant species to minimize interference with insect behavior during sampling. We recorded contacts between insects and flowers during 3-min periods at SB, 6-min periods at CM and 7.5-min periods in both sites on Lanzarote. Longer censuses were carried out in the last three locations because of the lower number of simultaneous species in bloom than at SB. During each census, we recorded: (a) identity of the flowering plant species, (b) number of open flowers of each individual plant observed; (c) identity of each flower-visitor (species name if possible or morphotype otherwise); (d) number of individuals of each species visiting flowers, and (e) number of flowers visited by each individual flower visitor. Insects that could not be identified in the field were collected for further identification by taxonomists.

Total time spent censusing flower-visitor interactions was 49:39 h at SB, 84:45 h at CM, 56:38 h at CB and 80:53 h at LC. Differences in total observation times among sites were a result of differences in the duration of the flowering period of their constituent plant species.

### Construction of quantitative flower-visitation networks

Quantitative interaction networks were constructed using flower visitation rate (FVR) as interaction weight, a measure of the intensity of mutual interaction strength between partners. The FVR of species was calculated as the number of flowers contacted by each flower-visitor species during a census, standardized by number of flowers observed, total census time per plant species, and specific flower abundance [33].

Additionally, in order to analyze the differences in partners’ traits and the temporal dynamics of the most generalized, specialized, selective and opportunistic species in each community along the season, we built 16 temporal interaction networks (“temporal snapshots”) [34], one per month in each community. Temporal snapshots reflect network structures more realistically than full-season networks as they include only those species that coexist in time.

### Specialization indexes

Both *L* and *d’* were calculated for all species in the four season networks, i.e. with data from the entire season (367 flower-visitors and 150 plant species), using the *bipartite* package version 1.17 [35] run in *R 2*.*11*. In order to work with more reliable specialization indices, we excluded from the dataset those plant species that had been censused less than 30 min in total as well as those flower-visitors observed less than five times. With this information, for each community we chose the 10 most generalized (highest *L*) and the 10 most specialized (lowest *L*) species (five plant and five flower-visitor species) which were present at least in two temporal networks. Likewise, we chose the 10 most opportunistic (lowest *d’*) and the 10 most selective (highest *d’*) species in each community. In some cases, the same species was in two categories, e.g. when it was both among the most generalized and among the most opportunistic. In total, we selected 117 species for which we obtained their partner profile (see S1–S4 Tables).

### Species and partners’ traits

We evaluated the evenness of partners’ abundances by calculating Pielou's measure of species evenness, i.e. ***J'*** = *H*'/ln(*S*) [36]. This evenness index varies between 0 and 1. The lower the variation among partner’s abundances, the higher *J'* is. For species with only one interacting partner we consider J' *= 1*.

Moreover, we estimated the abundance of a species in a community context, and categorized species as following:

**Highly abundant (5):** We look at the number of flowers that the most abundant species have on each community each month. Then, we see if another species has an abundance above 80% (because we have five categories). If so, we assign the same category rank.

**Abundant (4):** In the next step we do the same as before but having excluded the highly abundant species. We look at the number of flowers that the most abundant species have and see if another species has an abundance above 75% (because we have now four categories). If so, we assign the same rank category.

**Common (3):** Again, with the remaining dataset (i.e. excluding the species in the two previous categories) we look at the more abundant species and see if another species is above 66% (three categories left). If so we assign the same rank category.

**Low abundant (4):** We do the same as for the other four previous categorizations, but in this case we look for species above 50% in their abundance. If so, we assign the same rank category. The rest of them (below 50%) are considered **Scarce (5)**.

We further categorized each species into families -in the case of plants- and into functional groups -in the case of insects. In the latter case, we considered a total of 10 functional groups depending on insect size and foraging behavior: large bees (> 1cm), small bees (< 1cm), flies, hoverflies, beetles, wasps, butterflies, true bugs, ants and others (grasshoppers and acari).

### Statistical analysis

To test for differences in plants and flower-visitors partners’ traits (functional richness, rank abundance and evenness of abundances), we conducted general linear mixed models (GLMMs) with trait as dependent variable (one at a time), specialization index as fixed factor, and month nested within community as random factor. Separate models were built for the two types of specialization indexes, those considering linkage level (generalized/specialized) and those considering selectiveness level (opportunistic/selective).

Secondly, we tested if partner’s traits changed throughout the season depending on the degree of specialization and the degree of selectiveness or if species were constant in their partner’s traits. We conducted GLMMs for each specialization index separately (generalized, selective, opportunistic and selective) with trait as dependent variable (one at a time), using month as fixed factor and month nested within community as random factor.

The Tukey’s test (with the glht; 'many-to-one comparison procedure' [37]) was used to test for differences across groups when significant differences in a given factor were detected. For the analyses that included linkage level as fixed factor, flower-visitors’ functional richness was log transformed for a better fit with residual normal distribution. All analyses were performed using packages *lme4* [38] and *multcomp* [39] in *R 2*.*11*.

## Results

### Differences in partners’ traits

**Fig 2:**
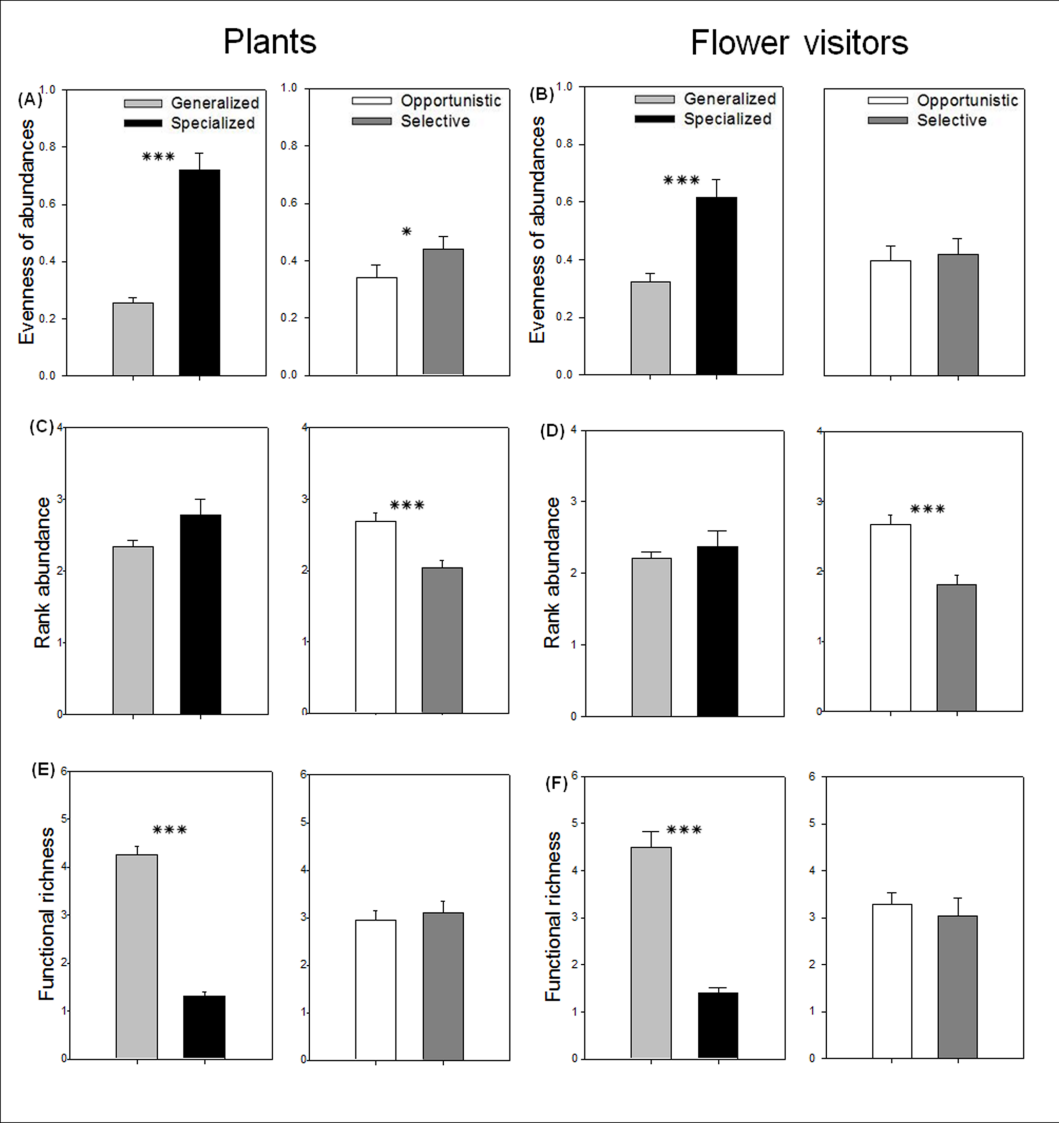
Mean (± SE) of partners’ traits of generalized (grey), specialized (black), opportunistic (white) and selective (dark grey) species. (a-b) Evenness of partner’s abundance, (c-d) partner’s rank abundance (5 = highly abundant and 1 = scarce), (e-f) partner’s functional richness. For each parameter, significant differences between categories are indicated by * (P < 0.5) and *** (P < 0.001).

As expected, generalized species showed lower evenness of partners’ abundance (plants: z = 7.523, p < 0.001, Fig 2A and flower-visitors: z = 4.768, p < 0.001, Fig 2B) and greater partners’ functional richness (plants: z = -12.9, p < 0.001, Fig 2E and flower-visitors: z = -10.55, p < 0.001, Fig 2F) than specialized species. However, rank abundance of partners did not differ between them (Fig 2C and 2D). These findings suggest that generalized species interact with many different species showing great variability in abundance among them whereas specialized species interact only with few but common species (very high evenness of abundances and the highest rank, near 3; i.e. with common species).

By contrast, opportunistic and selective species differed in their partner’s rank abundance (plants: z = 4.781, p < 0.001, Fig 2C and flower-visitors: z = 4.563, p < 0.001, Fig 2D). Moreover, the evenness of abundances was lower between the partners of opportunistic plant species than those of selective plant species (z = 2.061, p < 0.05, Fig 2A). However, both showed a similar partner’s functional richness (Fig 2E and 2F), indicating that they interact with a similar diversity of partners. These results show that opportunistic species interact mainly with diverse but abundant species whereas selective species interact with diverse but scarce species.

### Seasonal patterns of species partners’ traits

**Fig 3:**
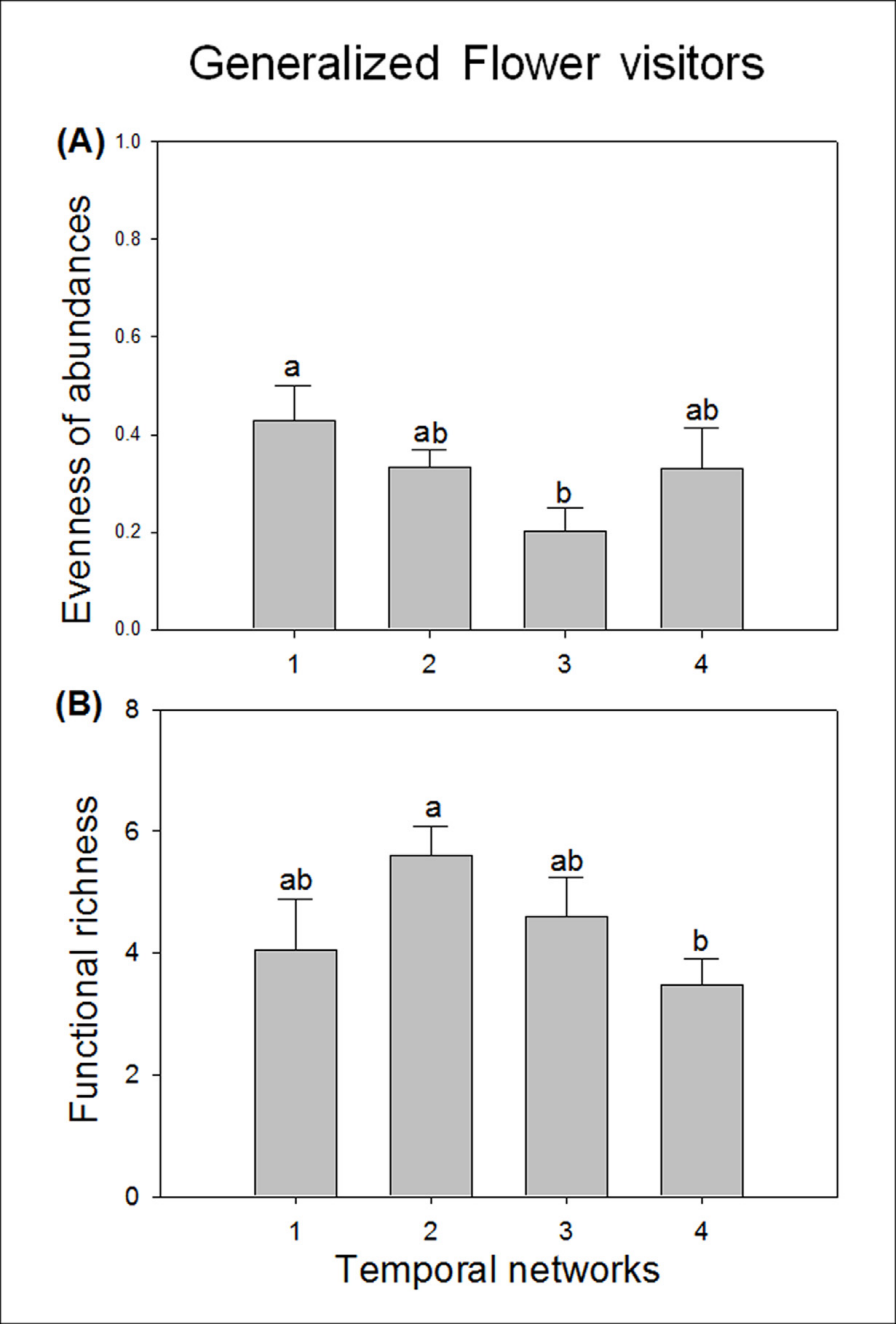
Changes across the season (temporal networks) in partners’ traits of generalized flower-visitor species. Each temporal network represents one month of the season. Mean (± SE) of: (a) evenness of partners’ abundances and (b) partners’ functional richness. For each parameter, values with the same letter are not significantly different from each other.

The evenness in flower abundance and family richness of partners used by generalized flower-visitor species varied significantly across the season. However, the rank abundance of their partners did not vary across the season. By contrast, specialized species were more constant through time. Generalized insect species visited flowers with the greatest variability in abundance during the third month of the season (significant differences where found only between the first and the second month: Fig 3A; z = -2.994, p < 0.05). However, it was the second month when they interacted with the largest number of partner’s families, though significant differences where found only between the second and the last month (Fig 3B; z = -2.738, p < 0.05). The highest number of plant families in each community in three of the four communities was found also during the second month of the season (Table 1); however, the month showing highest evenness on flower abundances varied among the four communities (Table 1). Neither opportunistic nor selective flower-visitor species varied significantly in any of the three partners’ traits, i.e. they consistently interacted with partners with the same traits across the season.

**Table 1:** Descriptive measures of each study area per month.

			Plants			Flower visitors		
Study area	month	total interactions	N° species	family richness	heterogeneity of abundances	N° species	functional group richness	heterogeneity of abundances
**Caletón Blanco**	1 (January)	158	10	8	0.4375	24	8	0.4187
** **	2 (February)	153	14	10	**0.4636**	24	8	**0.4298**
** **	3 (March)	**431**	14	**11**	0.4220	23	7	0.1808
** **	4 (April)	257	**15**	10	0.3645	**28**	**9**	0.4046
**Las Conchas**	1 (January)	**628**	15	11	0.4224	35	8	0.5153
** **	2 (February)	411	19	**13**	**0.4258**	30	**9**	0.2940
** **	3 (March)	428	**21**	11	0.2928	32	8	0.4919
** **	4 (April)	459	19	10	0.1077	**36**	**9**	**0.5184**
**Cala Mesquida**	1 (April)	148	17	7	0.2598	26	7	0.2822
** **	2 (May)	786	**31**	**13**	0.3139	57	**10**	0.3780
** **	3 (June)	**829**	28	10	0.2485	**78**	**10**	**0.4063**
** **	4 (July)	279	9	3	**0.3340**	35	8	0.3951
**Son Bosc**	1 (April)	814	33	20	**0.4132**	42	9	0.2195
** **	2 (May)	**1233**	**48**	**24**	0.3110	**74**	9	0.3480
** **	3 (June)	871	40	20	0.0474	70	**10**	0.4087
** **	4 (July)	277	21	13	0.0015	41	8	**0.5110**

The highest value of each measurement is indicated in bold.

Regarding plants, no significant temporal differences were found between either generalized and specialized or opportunistic and selective species in their partner traits.

## Discussion

Our findings support the prediction that the partners of generalized species have the highest functional richness and are highly heterogeneous in abundance, i.e. generalized species visit (in the case of animals) or are visited (in the case of plants) by a wide range of different partners being abundant or scarce and belonging to different pollinator functional groups and plant families. Also as expected, specialized species tended to visit or be visited mostly by common species belonging to the same plant family or pollinator functional group, thus avoiding or decreasing the extinction risk of losing mutualistic interactions due to the different drivers of global change [40]. This finding also supports previous results from studies that have shown that reciprocal specialization between species is rather rare and that interactions among mutualistic partners are highly asymmetric, a pattern that contributes to increase community nestedness [26, 27]. This pattern has shown to decrease interspecific competition in those communities with highest niche overlap, nested or fully connected networks [41, 42].

We also found support for the prediction that both opportunistic and selective species have a high functional richness of partners. This result is particularly interesting as it shows that selective species, considered so far to be most vulnerable to disturbances, might be maintained in the community by interacting with such a wide array of partners. Interacting with scarce but functionally rich partners might actually be a ‘strategy’ of selective species to avoid competition for abundant resources and ensure their maintenance in the community. Curiously, opportunistic species showed less functional richness of partners than generalized species, what illustrates the difference between the two types of specialization indices. Generalized species visit or are visited by species regardless of their abundance, but the partners of opportunistic species are always abundant species. Some of the plant families or pollinator functional groups in the study communities do not have abundant species. This is the case of plant families such as Aizoaceae, Malvaceae and Papaveraceae or of pollinator functional groups such as true bugs and butterflies. Moreover, selective species showed higher functional richness of partner’s than specialized species. This is because selective species mainly visit or are visited by scarce partners, and most plant families and pollinator functional groups are actually very scarce species in our communities whereas, as mentioned above, specialized species interact mainly with common species of a lower number of plant families or insect functional groups.

Some studies have shown a high variability in species and interaction turnover within the same season and across seasons [17, 28, 29, 31] but rather little is known about the mechanisms of such changes. Interaction turnover may occur because species change preferences responding to the abundance of their partners, or because a new species suddenly appears in the community, i.e. interaction turnover may be due to temporal dynamics in the selection of partner’s traits. The temporal fluctuation in species and interactions could be cushioned if one lost species is replaced by another with the same or similar traits [31]. Our results actually show that this is the case for almost all species chosen for our study. Despite new flower-visitor species appear and disappear and abundances change, all selected plants (generalized, specialized, opportunistic and selective) were very constant in their partner’s traits across the season. Similarly, specialized, selective and opportunistic flower-visitor species did not change their partners’ use across time. By contrast, the generalized flower-visitor species exhibited shifts through time in their partners’ traits depending on the species composition in the community across the season. The diet breadth of generalized pollinators is known to be a flexible trait, resulting in part from adaptation of foraging choices to resource availability [42, 43].

Although our findings are based on a single season, we believe they are well informative on the microstructure of pairwise interactions, as we analyze flowering species and flower-visitor species that coexist in time (i.e. “forbidden links” due to phenological mismatching between partners are reduced to minimum). We also reduced biases due to sampling effort by censusing (each census day) the same amount of time both rare and abundant species and by using an interaction weight that accounts for both the number of observed flowers in each census as well as the flower abundance of each plant species observed [33]. Using the total number of individuals observed visiting flowers to estimate flower-visitor abundance is arguably limited but the best estimate we can obtain. Even so, the results for flower-visitors were similar to those obtained for plants, for which we did have independent estimates of abundance, supporting the general trends we found. Nevertheless, to generalize on our findings, further exploration of other communities and with data from more seasons might be required. Moreover, other relevant partners’ traits, such as the type of reward offered by the flowers [44], floral display and floral and insect size and form [45, 46], color [47] or scents [24] should be considered if we are to unravel the different mechanisms influencing the microstructure of a pollination network.

The concept of specialization in plant–pollinator systems is inevitably connected to the notion of extinction cascades in natural ecosystems, i.e. the idea that if the pollinator of a specialized plant becomes extinct, then the plant is bound to follow and vice versa. However, we have shown that the most specialized species interact with abundant species in the community, what gives robustness to the pollination networks in the face of disturbances and species loss. By linking to common species, specialized species contribute to increase nestedness, which is important to network robustness and stability to species extinctions [13, 48–50]. Likewise, selective species visit or are visited by partners belonging to different families or functional groups and this may provide them with a higher resistance to cope with partners’ extinction.

These considerations are therefore important in the context of global change. Based on our results, we predict that the great majority of both plants and flower-visitors will find new interaction partners with similar traits to those that could vanish, i.e. we predict that the probability of species re-wiring is high and extinction cascades may not occur as rapidly as previously thought in these types of communities [51–52]. However, studies that incorporate measures of pollinator services are badly needed to determine how this rewiring could affect plant fitness and pollinator-mediated selection.

## Supporting Information

S1 Table Information on plant species chosen of each network. Here we indicate: zone (SB = Son Bosc, CB = Caletón Blanco, LC = Las Conchas and CM = Cala Mesquida), family, species code (cod.sp), linkage level (*L*), level of selectiveness (*d’*) and the grade of specificity (generalized, specialized, opportunistic or selective species). For this study were chosen five of the most generalized (highest *L*), five of the most specialized (lowest *L*), five of the most opportunistic (lowest *d’*) and five of the most selective (highest *d’*) plant species in each community, present at least in two temporal networks.
(DOC)

S2 Table Information on flower-visitor species chosen of each network. Here we indicate: zone (SB = Son Bosc, CB = Caletón Blanco, LC = Las Conchas and CM = Cala Mesquida), functional group, species code (cod.sp), linkage level (*L*), level of selectiveness (*d’*) and the grade of specificity (generalized, specialized, opportunistic or selective species. For this study were chosen five of the most generalized (highest *L*), five of the most specialized (lowest *L*), five of the most opportunistic (lowest *d’*) and five of the most selective (highest *d’*) flower-visitor species in each community, present at least in two temporal networks.
(DOC)

S3 Table Detailed information of our Plant dataset. (DOC)

S4 Table Detailed information of our Flower-visitor dataset. (DOC)
