# Supplementary material for: Contrasting Partners’ Traits of Generalized and Specialized Species in Flower-Visitation Networks
Source: PLoS One. 2016 Mar 3;11(3):e0150824. doi: 10.1371/journal.pone.0150824 (PMC4777429; doi:10.1371/journal.pone.0150824)
Supplement: S4 Table — (DOC) [file pone.0150824.s004.doc]

**Table S4a**

**Table S4b**

**Table S4c**

**Table S4d**
